# Influence of Aggregation and Route of Injection on the Biodistribution of Mouse Serum Albumin

**DOI:** 10.1371/journal.pone.0085281

**Published:** 2014-01-22

**Authors:** Grzegorz Kijanka, Malgorzata Prokopowicz, Huub Schellekens, Vera Brinks

**Affiliations:** 1 Department of Pharmaceutics, Utrecht Institute for Pharmaceutical Sciences (UIPS), Utrecht University, Utrecht, The Netherlands; 2 Department of Innovation Management, Utrecht University, Utrecht, The Netherlands; The Ohio State University, United States of America

## Abstract

Protein aggregates are a major risk factor for immunogenicity. Until now most studies on aggregate-driven immunogenicity have focused on linking physicochemical features of the aggregates to the formation of anti-drug antibodies. Lacking is however, basic knowledge on the effect of aggregation on the biodistribution and clearance of therapeutic proteins *in vivo*. The aim of current study was to get insight into the effect of aggregation on biodistribution in mice using different routes of administration. Fluorescently labeled stressed and unstressed mouse serum albumin was injected via different routes in mice and detected via *in vivo* fluorescence imaging up to 48 hrs post-injection. We found that biodistribution of stressed MSA significantly differed from its unstressed counterpart. Subcutaneous and intramuscular administration resulted in accumulation of protein at the site of injection, from which clearance of stressed MSA was considerably slower than clearance of unstressed MSA. Upon intravenous and intraperitoneal injection of stressed MSA, fluorescent “hotspots” were observed in the spleens, livers and lungs. Further and more detailed examination of biodistribution after intraperitoneal injection showed higher fluorescence in most of tested organs suggesting more efficient diffusion and/or lymphatic uptake from peritoneum of unstressed MSA than the stressed formulation.

## Introduction

Therapeutic proteins have revolutionized the therapy of many diseases like multiple sclerosis, rheumatoid arthritis, Crohn's disease and many others. Unfortunately, therapeutic proteins are immunogenic and cause the production of anti-drug antibodies (ADA) in some patients. These ADA can decrease the treatment efficiency and can lead to severe side effects [1]–[3].

Among many risk factors that could induce the production of ADA protein aggregates seem to be critical. An increasing number of reports link the presence of protein aggregates in the formulated product to an increased risk of ADA formation [4]–[9]. Various physicochemical features including aggregate size, molecular weight , composition and rigidity have been studied to determine, which are critical in immunogenicity [6]–[8]. However, data on aggregates' fate after their administration into patients is very limited. Filipe et al. showed that incubation of human monoclonal IgG aggregates in plasma for 24 hrs resulted in alteration of the total number of aggregates, led to different aggregate size and changed their structure [10]. These results indicate that aggregates can undergo significant modifications after coming in contact with biological fluids.

Many reports, both from clinical and animals studies, have shown that the route of injection might have a significant impact on immunogenicity of therapeutic proteins [11]–[14]. One of the explanations of this phenomenon is distinct biodistribution of drugs after administration via different routes [15], [16]. However, studies comparing biodistribution of (aggregated) proteins administered via different routes are lacking. Since the physicochemical characteristics of aggregates and monomers differ significantly, it seems likely that the biodistribution of these species is also different. In fact, existing literature seems to suggest differences in biodistribution of protein monomers and aggregates. For example, it has been shown that uptake of proteins after subcutaneous (SC) injection occurs mainly via lymphatic transportation, which can carry macromolecules and particulates up to 100 nm in diameter [17]. However, as aggregates often exceed this size, one could imagine that clearance of aggregates from the injection site upon SC administration will be slower than that of monomers. Decomposition of protein aggregates might be necessary prior to their removal. One could also hypothesize that after intravenous (IV) injection protein aggregates are cleared from circulation by the reticuloendothelial system as it has been shown for liposomes [18]. However, these hypotheses need to be confirmed.

This report describes a series of experiments designed to study the biodistribution of aggregated proteins after administration in a mouse model. In order to obtain an autologous system mimicking human situation we used mouse serum albumin (MSA) as a model protein, which was labeled with an infrared fluorescence probe to allow detection *in vivo and ex-vivo*. In the first experiment we administered unstressed or stressed (aggregated) MSA via four different injection routes: intraperitoneal (IP), IV, SC or intramuscular (IM) and assessed fluorescence over a period of 48 hours. In a follow up study we determined in more detail the biodistribution of unstressed and stressed MSA over time after IP injection.

## Materials and Methods

### 1. Animals

All testing was performed according to the EU Directives 86/609/EEC and 2010/63/EU. The work protocols have been approved by Utrecht Animal Experiments Committee (DierExperimentenCommissie Utrecht, permission numbers: DEC no. 2012.II.04.064 and 2012.II.07.099). All procedures were kept to minimum to limit animals' discomfort. Animals were sacrificed by decapitation preceded by isoflurane anesthesia.

FvB/N females (5–6 weeks old) were purchased from Charles River (The Netherlands). Animals were housed in standard perspex cages with free access to food and water (acidified). To reduce food-induced fluorescence in the intestinal track, animals received chlorophyll free diet (Harlan Laboratories, The Netherlands). In order to eliminate autofluorescence caused by the fur, mice were completely shaven and treated with Veet cream (local pharmacy) to remove remaining hair.

### 2. Protein labeling and stressing

MSA (Sigma Aldrich, the Netherlands) was reconstituted in phosphate buffered saline (PBS) to a concentration of 5 mg/ml. It was labeled with Alexa Fluor 700 carboxylic acid succinimidyl ester (Invitrogen, the Netherlands) according to the manufacturer protocol. The molar ratio of dye to protein was 10∶1. After labeling, the free dye was removed by overnight dialysis against PBS. The degree of protein labeling was determined according to the manufacturer's protocol and was equal to 3 Alexa700 dye molecules per one MSA molecule.

Stressed MSA-Alexa700 was acquired via metal catalyzed oxidation. First, MSA-Alexa700 conjugates were dialyzed overnight (O/N) against 50 mM HEPES, 100 mM KCl, 10 mM MgCl_2_, pH 7.4 (oxidation buffer). After dialysis MSA was diluted to a concentration of ∼1.2 mg/ml and the oxidation procedure was initiated by adding FeCl_3_ and ascorbic acid to final concentrations of 100 mM and 2.5 mM, respectively. The solution was kept at 37°C for one hour, after which the oxidation reaction was terminated by the addition of EDTA to a final concentration of 1 mM. Afterwards, stressed MSA-Alexa700 was again O/N dialyzed against PBS. Protein concentration was determined by a MicroBCA® assay according to the manufacturer's protocol (Thermo Fisher Scientific, The Netherlands). MSA-Alexa700 solutions (stressed and unstressed) were diluted to a final concentration of 1 mg/ml, aliquoted and stored at 4°C (short term storage, <week) or −20°C (long term storage, up to 4 weeks).

### 3. Characterization of unstressed and stressed MSA

#### Visual inspection

Stressed samples were visually inspected to determine the presence of visible particles. Unstressed MSA-Alexa700 formulation was used as a reference.

#### Size-Exclusion Chromatography (SEC)

SEC was performed on a Waters 2699 Aliance (Waters, USA) equipped with a WATERS 2487 Dual λ absorbance detector and a WATERS 2475 Multi λ fluorescence detector. Proteins were separated on a Superdex 200 column (GE Health Care Life Sciences, Belgium). The elution buffer was composed of 50 mM phosphate buffer, 200 mM NaCl and pH 7.4. Before analysis both the buffer and the MSA-Alexa700 samples were filtered through a 0.45 µm Nylon filter. A volume of 100 µl stressed or unstressed MSA-Alexa700 solution was applied to the column and the separation was performed with a flow rate of 0.5 ml/min. UV detection was performed at a wavelength of 280 nm (A280) and fluorescence was induced at 702 nm and detected at 720 nm (F720).

In order to calculate the amount of free dye in the formulations the area under the curve (AUC) was calculated for the F720 signal. The percentage of oligomers, monomers and fragments of MSA present in both stressed and unstressed formulations were calculated for the AUC measured at A280. To calculate the recovery of the stressed MSA-Alexa700 after filtration, the AUC of stressed MSA-Alexa700 was compared with the AUC of the unstressed MSA-Alexa700, which was referred to as 100%.

#### Sodium Dodecyl Sulfate Polyacrylamide Gel Electrophoresis (SDS-PAGE)

The SDS-PAGE analysis was performed with the X-Cell Sure Lock^@^ Mini-Cell system (Invitrogen, the Netherlands). 0.5 µg or 1 µg of stressed and unstressed MSA-Alexa700 were loaded on the NuPAGE® 4–12% Bis-Tris Mini Gels of 1 mm thickness (Novex®, Invitrogen, the Netherlands). Protein samples were separated under non-reducing conditions at 130 V for 60 minutes. Proteins were visualized by silver staining and by detection of fluorescence using the infra-red imager Odyssey (LiCore, Germany).

#### Dynamic Light Scattering (DLS) and NanoParticle Tracking Analysis (NTA)

DLS measurements were performed on a Malvern ALV CGS-3 goniometer (Malvern Instruments, Malvern, UK) coupled to an LSE-5003 autocorrelator and a He\Ne laser (25 mW, output power, λ = 632.8 nm). Data were obtained at 25°C and the laser was operating at an 90° angle. Data were captured by the ALV correlator software (Malvern, UK) and analyzed using the DTS (Nano) software (Malvern, UK).

In addition, both stressed and unstressed MSA-Alexa700 were analyzed for the number of particles using the NanoSize LM14 (NanoSight Ltd., UK) equipped with an EMCCD/Andor camera and a 532 nm laser. Particle count and size distribution were analyzed by three 60 s measurements.

### 2. *In vivo* studies

#### Experiment 1: Biodistribution of unstressed and stressed MSA upon injection via different routes

A volume of 50 µl (50 µg) of unstressed or stressed MSA-Alexa700 was injected via one of following routes: IP, IV, IM (right hind leg) or SC (neck), (n = 5). Fluorescence was measured by the BioSpace Photon Imager™ (Biospace Lab, France) before injection, directly post injection (p.i.), every 10 min within the first hour p.i. and after 3, 5, 8, 24, and 48 hrs p.i. The fluorescence was excited at 696 nm and emission was measured at 720 nm for 10 s. Using the autofluorescence measured for all animals before the injection of MSA, a fluorescence threshold value was determined at 10 counts per s. Also, regions of interest (ROIs) were drawn around the injection site of animals treated SC and IM. The maximal fluorescence measured at the neck (corresponding to SC injection site) and the right hind leg (corresponding to IM injection site) of mice administered IP and IV, was used to set the fluorescence threshold for these ROIs (≥70 counts per s).

After 48 hours all mice were euthanized and following organs and tissues were collected for *ex vivo* analysis of fluorescence: bladder, spleen, kidneys, liver, lungs, heart, skin (from site of injection of animals injected SC and IM), muscle (IM) and plasma. Organs were stored at −80°C and plasma at −20°C prior to *ex vivo* analysis.

*Ex vivo* fluorescence of spleens, livers and lungs, was measured on an infra-red imager (Odyssey, LiCore, Germany). The distribution of MSA-Alexa700 conjugates in all collected organs and tissues was determined using an adopted method for the measurement of anti tumor nanobody biodistribution described by Oliveira et al [19]. Briefly, organs were first weighed and homogenized in a radioimmunoprecipitation buffer (RIPA, 50 mM Tris, 150 mM NaCl, 0.1% SDS, 0.5% sodium deoxycholate and 1% Triton X 100) supplemented with protease inhibitors (Sigma, the Netherlands). Homogenized solutions were transferred onto 96-well plates (Greiner bio one, The Netherlands) and fluorescence was measured on the infra-red imager. The concentration of MSA-Alexa700 in every organ was calculated from a standard curve created by serial, two-fold dilutions of known concentrations of unstressed or stressed MSA-Alexa700 conjugates in RIPA buffer.

#### Experiment 2: Detailed biodistribution of unstressed and stressed MSA upon IP administration

Mice (n = 5 per group) were injected IP with either unstressed or stressed MSA-Alexa700. Before and after the injection the syringes were weighted to precisely determine the injected dose of MSA-Alexa700 conjugates. *In vivo* fluorescence was assessed before injection, directly p.i. and before euthanasia (15 min, 1, 3, 8 and 24 hrs p.i) using the BioSpace Photon Imager™. Mice were euthanized by decapitation under isoflurane anesthesia. Afterwards, following organs and tissues were collected for *ex vivo* analysis: blood, urine, spleen, liver, kidney (left), stomach, small and large intestine, urinary bladder, muscle and skin (from right hind leg), lung, thymus, heart and brain.

*Ex vivo* fluorescence measurements and analysis of homogenized tissues and organs was performed as described above.

### 3. Statistical analysis

All data were first assessed for the normal distribution using the Shapiro-Wilk test. Because data from the first experiment 1 was not normally distributed, a Mann-Whitney non-parametric test was used to assess differences between *in vivo* fluorescence of the skin and muscle areas (ROIs) of animals injected IM and SC with either stressed or unstressed MSA-Alexa700. The same test was also used to analyze the difference in *ex vivo* MSA-Alexa700 signal between the different organs collected from mice treated with either stressed or unstressed MSA-Alexa700. To compare the difference in *ex vivo* MSA-Alexa700 signal between the organs collected using different injection routes a Kruskal-Wallis test was employed.

Data obtained during the experiment 2 was also not normally distributed, therefore also here non-parametric tests were used for analysis. A Mann-Whitney test was used to assess the difference in overall fluorescent signal (cumulated for all time points) between organs from animals injected with either stressed or unstressed MSA-Alexa700. A Kolmogorov-Smirnov test was employed to check if the distribution of the fluorescence measured in different organs *ex vivo* differed over time between stressed and unstressed MSA treatment.

## Results

### 1. Characterization of unstressed and stressed MSA

#### Visual inspection

**Figure 1:**
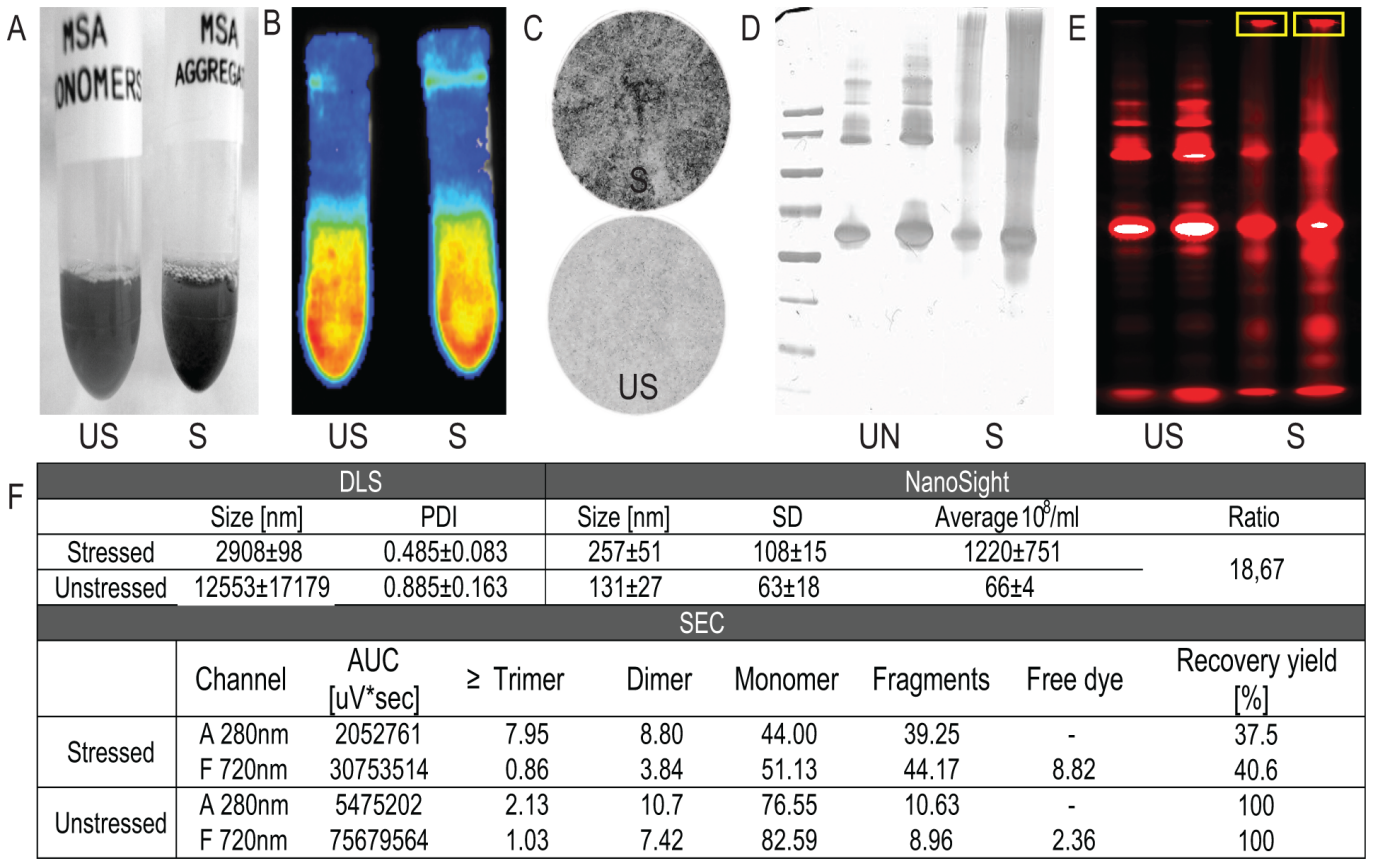
Physicochemical characterization of stressed and unstressed MSA. A) Photography of stressed and unstressed MSA-Alexa700 formulations. B) Fluorescence of unstressed (US) and stressed (S) MSA-Alexa700 formulations measured using an *in vivo* imager. C) Photography of 0.45 µm nylon filters surface after filtration of MSA-Alexa700 formulation prior to SEC analysis. D) Silver stained results of SDS-page analysis of the stressed and unstressed MSA-Alexa700 conjugates under non-reducing conditions. Samples order: marker, 0.5 µg and 1 µg of unstressed MSA-Alexa700, 0.5 µg and 1 µg of stressed MSA-Alexa700. E) Fluorescent analysis of protein separated on the SDS-page gel. Samples order: 0.5 µg and 1 µg of unstressed MSA-Alexa700, 0.5 µg and 1 µg of stressed MSA-Alexa700. The stressed formulation contains aggregates too big to enter the gel and stuck in the wells (yellow rectangles). F) Summarized results of DLS, NTA and SEC analysis of both stressed and unstressed MSA formulations.

Stressing the MSA-Alexa700 formulation via metal catalyzed oxidation resulted in the formation of insoluble, rapidly sedimending aggregates that were easily detectible by eye (Figure 1A).

#### Size exclusion chromatography

Before SEC analysis both stressed and unstressed MSA-Alexa700 solutions were filtered through a 0.45 µm nylon filter. Inspection of the filter revealed that a significant amount of MSA-Alexa700 aggregates was retained on the filter used for the stressed solution (Figure 1C). This observation was confirmed by the total AUC of stressed MSA-Alexa700, which showed a protein loss of ∼60% after filtration when compared with total AUC of unstressed MSA-Alexa700 (Figure 1F). SEC analysis revealed a reduced percentage of protein monomers in the stressed MSA-Alexa700 sample when compared to the unstressed MSA-Alexa700 formulation (76% unstressed vs 44% stressed formulation). The percentage of protein dimers was similar in both formulations (8.8% and 10.7%), but the percentage of trimers and higher molecular weight species was slightly increased in the stressed MSA-Alexa700 solution (7.9% vs 2.3%). Interestingly, SEC analysis revealed degradation of MSA-Alexa700 during the stress procedure since the amount of protein fragments in the stressed formulation was three fold higher compared to the unstressed formulation (30% vs 10%, respectively).

SEC analysis also allowed estimating the contents of free dye in the stressed and unstressed MSA-Alexa700 solutions. As shown in Figure 1F only 2.4% of the fluorescent signal originated from unbound dye in the unstressed formulation. In contrast, in the stressed MSA-Alexa700 the contents of free dye seemed to be higher (8.8%), however after correcting for the protein loss during filtration of this stressed solution the amount of free dye was estimated to be 3%.

#### Sodium Dodecyl Sulfate Polyacrylamide Gel Electrophoresis (SDS-PAGE)

Silver staining revealed the presence of multiple multimeric entities (up to the estimated weight of MSA tetramers) in the unstressed MSA-Alexa700 formulation. However, in this unstressed formulation monomers were the most abundant species (Figure 1E). Also, no aggregates or fragments were detected (Figure 1D). In contrast, the stressed formulation contained significant amounts of both MSA fragments and aggregates (Figure 1D). In fact, the stressed MSA-Alexa700 contained aggregates, which were too big to enter the gel (Figure 1E).

#### DLS and NTA

Both DLS and NTA analyses confirmed that the stressed MSA-Alexa700 formulation contained higher amount of aggregates compared to the unstressed formulation (Figure 1F). For the stressed MSA-Alexa700 DLS analysis revealed presence of particles with an average size of 2908±98 nm and a PDI of 0.485±0.083. For the unstressed formulation DLS analysis gave inconclusive results as the measured size varied from about 400 nm to 20.000 nm depending on the measurement, and the PDI values were very high (0.7 to 1). NTA measurements showed the presence of particles in both stressed and unstressed formulations with an average size of 257±51 nm (stressed) and 131±27 nm (unstressed). Moreover, NTA analysis showed an ∼18 fold increase in particle count in the stressed MSA-Alexa700 solution compared to the unstressed one.

### 2. *In vivo* studies

#### Experiment 1: Biodistribution of unstressed and stressed MSA upon injection via different routes

*In vivo* and *ex vivo* analysis of MSA-Alexa700 conjugates upon injection revealed significant differences in biodistribution between the stressed and non-stressed formulations.

#### Intravenous injection

**Figure 2:**
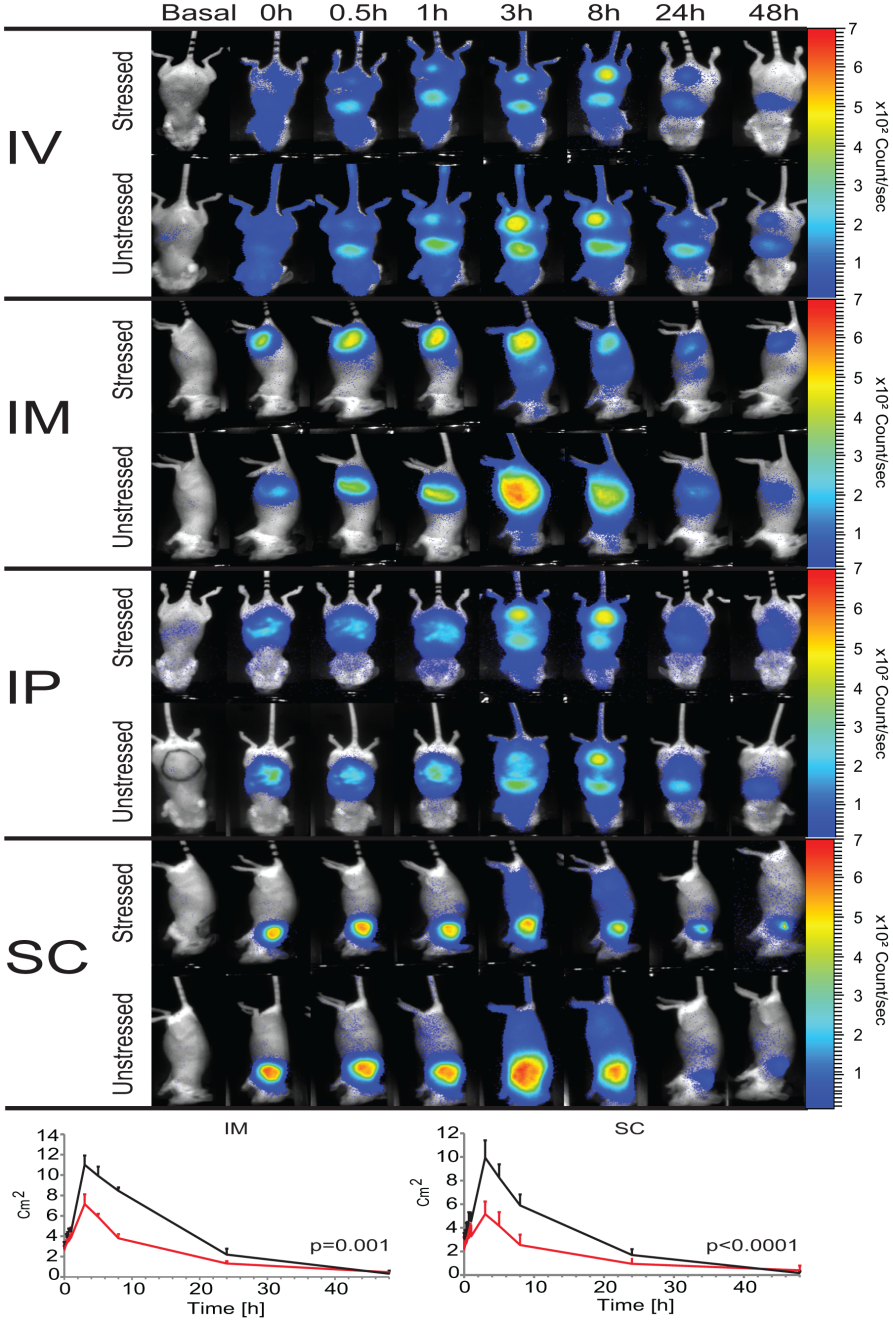
*In vivo* determination of distribution of unstressed and stressed MSA-Alexa700 upon injection via different routes. A) *In vivo* distribution of fluorescence signal from MSA-Alexa700 conjugates upon injection via IV, IM, IP and SC route in time. B) Change of size of ROIs drawn around the injection site of animals injected IM or SC. The black line shows results obtained in animals injected with unstressed MSA-Alexa700 and red from animals treated with stressed protein.

As shown in Figure 2 IV injection led to a rapid distribution of MSA-Alexa700 conjugates throughout the body in case of both, stressed and unstressed, formulations. At 10 minutes p.i. accumulation of the fluorescent signal was observed in the area of the liver and 20 min p.i. high fluorescence was seen in the area of the bladder. At 24 hrs p.i., mice injected with stressed MSA-Alexa700 displayed fluorescence only in the liver and bladder areas. In contrast, animals injected with unstressed MSA-Alexa700 displayed fluorescence at this time point in other parts of their bodies as well. Both *in vivo* and *ex vivo* analyses showed that IV injection resulted in similar accumulation of fluorescent signal in the livers of mice receiving either stressed or unstressed MSA-Alexa700. Moreover, IV administration resulted in the highest amount of fluorescence in the liver area of all injection routes studied in this experiment (p<0.001 for IV vs IP/IM/SC). The accumulation of MSA-Alexa700 48 hrs p.i. in other organs than the liver was negligibly low.

**Figure 3:**
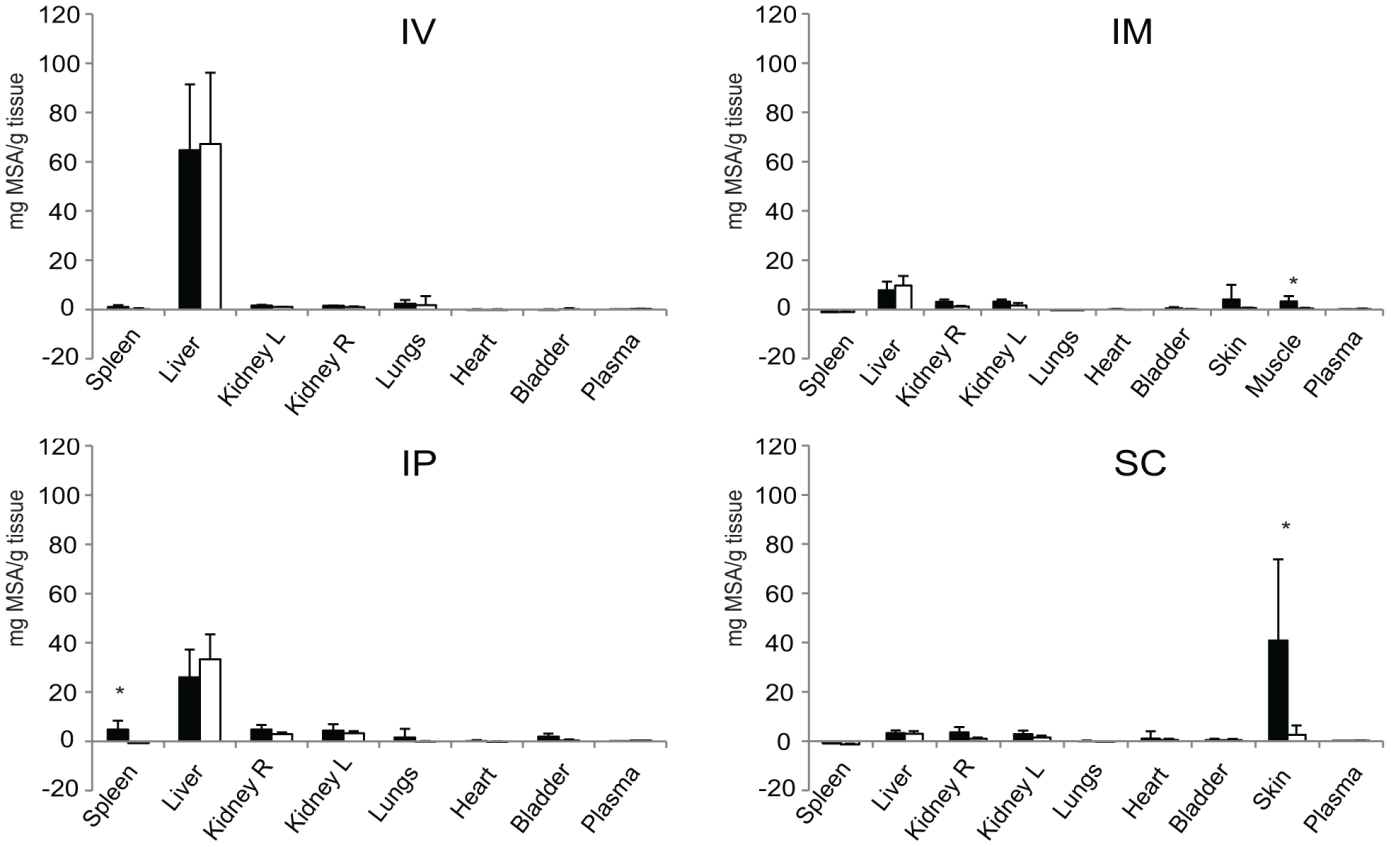
*Ex vivo* determination of distribution of unstressed and stressed MSA-Alexa700 48 hrs post injection. Distribution of fluorescence signal in different organs 48 p.i. via IV, IM, IP and SC routes. The bars show average accumulation of MSA-Alexa700 conjugates ± SD. The black bars show results obtained from animals injected with stressed protein and white bars indicate unstressed protein.

An interesting observation made during *ex vivo* measurements was the presence of “hotspots” with very high fluorescence in the lungs and spleens of mice treated with stressed MSA-Alexa700 (Figure 3). Organs collected from animals receiving the unstressed formulation did not show these “hotspots”.

#### Intramuscular injection

After IM injection, fluorescence could be measured at the site of injection until the end of the experiment (Figure 2). Moreover, the intensity of fluorescence measured *in vivo* at the site of injection 48 hrs p.i. seemed to be similar for mice treated with either stressed or unstressed MSA-Alexa700. However, *ex vivo* analysis revealed that mice treated with stressed MSA-Alexa700 displayed higher fluorescence signal in the muscle compared to mice treated with the unstressed formulation (p = 0.016). Also clear differences in the distribution kinetics at the site of injection were observed. Unstressed MSA-Alexa700 seemed to diffuse or be removed from the injection spot much faster than the stressed formulation. The spread of fluorescence signal (increase of ROIs) around the injection spot in animals receiving unstressed MSA-Alexa700 seemed to be faster and the surface area of ROIs was significantly higher compared to that of mice receiving the stressed formulation (p = 0.001). At 3 hrs p.i. fluorescence was found to be spread throughout the body, for both stressed and unstressed formulations. This body-wide fluorescence signal was still measurable 8 hrs p.i. *Ex vivo* analysis revealed low accumulation of fluorescent signal of stressed and unstressed MSA-Alexa700 in the liver 48 hrs p.i..

#### Intraperitoneal injection

Directly after IP injection of either stressed or unstressed MSA-Alexa700 conjugates a fluorescent signal was detectible in the whole peritoneum (Figure 2). No significant changes in the fluorescent signal (i.e. intensity or distribution) were observed within the first hour p.i.. At 3 hours p.i. fluorescence was detected throughout the body for both stressed and unstressed MSA-Alexa700 treatments, with a particular strong signal in the area of the liver and in the bladder. Up to 8 hrs p.i. fluorescence was detected throughout the body. 24 hours p.i. of stressed and unstressed MSA-Alexa700 a weak fluorescent signal could still be detected in the area of peritoneum and liver. However, this signal seemed to be more pronounced in animals receiving the unstressed formulation. At the end of experiment (48 hrs p.i.) residual fluorescence could be measured only in the liver area of mice receiving unstressed MSA-Alexa700. In contrast, mice injected with stressed MSA-Alexa700 displayed residual fluorescence in the area of liver and peritoneum.

**Figure 4:**
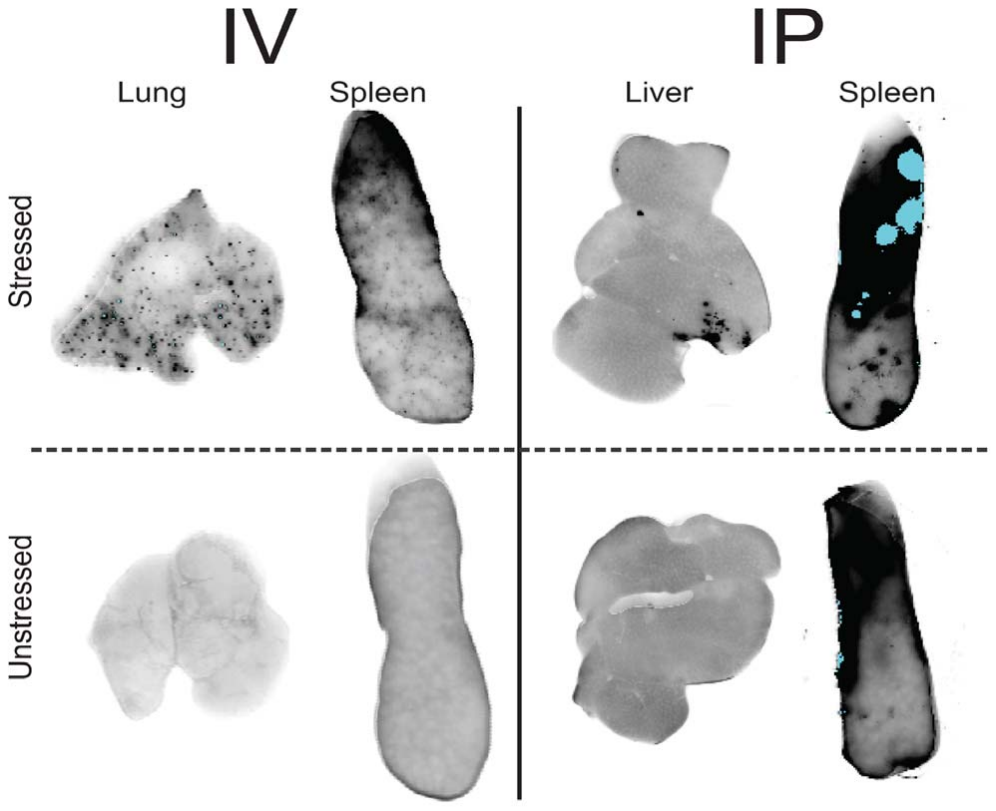
Stressed MSA-Alexa700 formed fluorescent “hot-spots” in lungs, livers and spleens of mice injected IP and IV. A) IV injection of stressed MSA-Alexa700 resulted in the formation of fluorescent “hotspots” in the lungs and spleens. B) In livers and spleens of animals IP injected with stressed protein clumps of highly fluorescent signal were observed.

*Ex vivo* analysis showed highest fluorescence signal in the isolated livers (Figures 3), with similar intensity in mice treated with stressed and unstressed formulations. However, in livers and spleens isolated from the mice injected with stressed MSA-Alexa700 “hotspots” of high fluorescence were detected. In contrast, in mice treated with unstressed MSA-Alexa700 no “hotspots” were observed in either the liver or the spleen and the fluorescent signal was equally distributed throughout those organs (Figure 4). Moreover, the total fluorescence signal in spleens isolated from mice treated with stressed MSA-Alexa700 was higher than the signal from mice treated with the unstressed formulation (p = 0.001).

#### Subcutaneous injection

SC injections resulted in similar to IM injections distribution pattern of fluorescent signal . After SC injection, the spread of fluorescent signal from the injection site seemed to be faster in mice treated with unstressed than with stressed MSA-Alexa700. However, in contrast to the IM route of injection, a clear difference in the intensity of fluorescence at the site of injection between stressed and unstressed formulations was still visible 48 hrs p.i.. *Ex vivo* analysis confirmed a significantly higher amount of MSA-Alexa700 at the site of injection (skin) of animals receiving the stressed formulation (p = 0.045).

#### Experiment 2: Detailed biodistribution of unstressed and stressed MSA upon IP administration

To determine the biodistribution over time after IP administration animals were treated with stressed or unstressed MSA-Alexa700 conjugates and euthanized at designated time points p.i.. Selected organs and tissues were collected for the *ex vivo* analysis. The *in vivo* and *ex vivo* measurements showed that some animals were injected incorrectly as shortly after the injection the fluorescent signal was not equally distributed throughout the peritoneum and post mortem analysis revealed presence of injection solution in the intestines or stomach of these mice. These mice were therefore excluded from the analysis.

**Figure 5:**
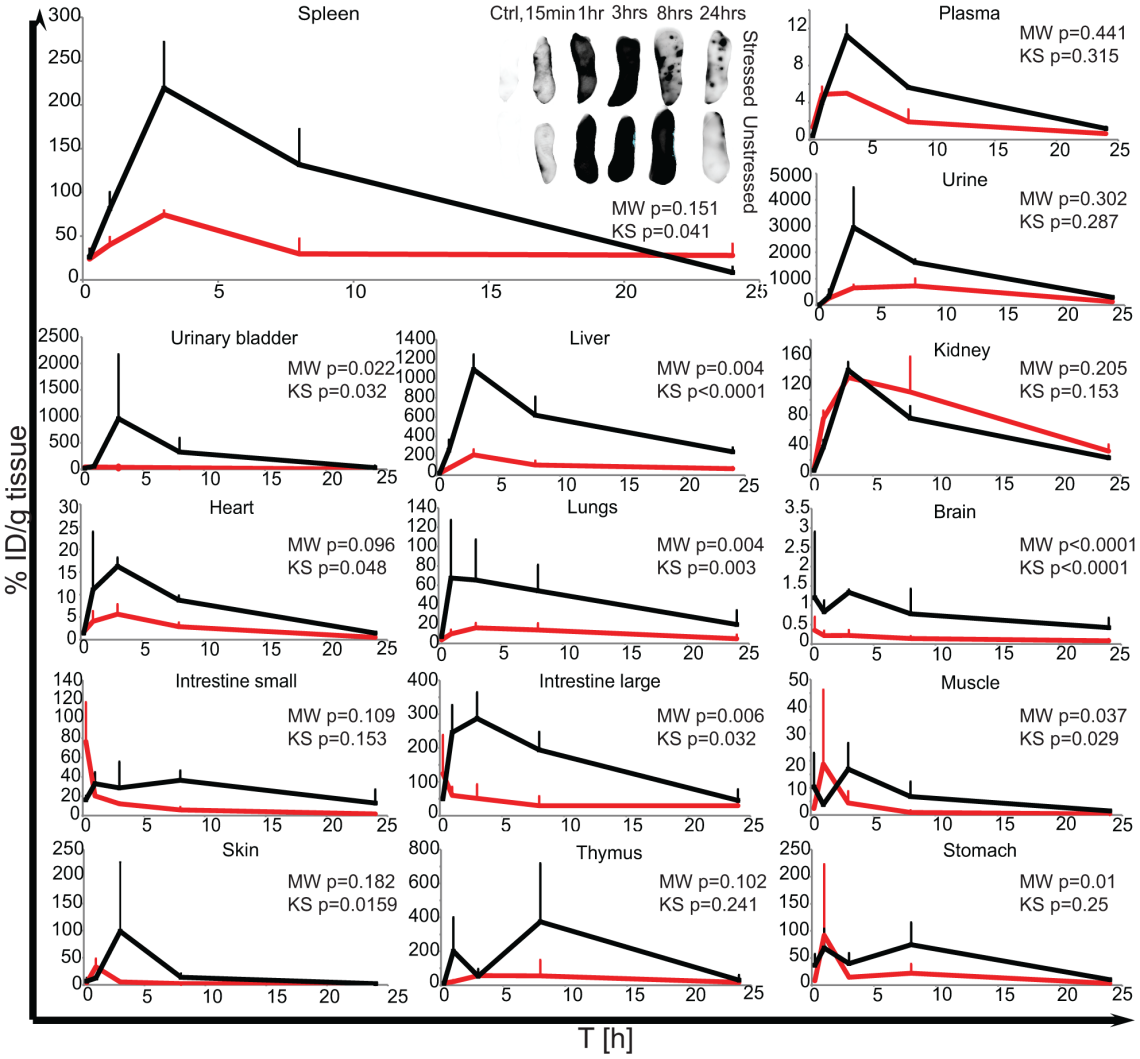
Detailed biodistribution over time of unstressed and stressed MSA-Alexa700 upon IP injection. The biodistribution of the MSA-Alexa700 over time in animals IP injected with stressed (red line) and unstressed (black line) formulations. The accumulation of fluorescent signal is expressed as percent of injected dose per gram of tissue (% ID/g tissue) or percent of injected dose per ml of plasma or urine (% ID/ml).

*In vivo* fluorescence measured up to 24 hrs p.i. was found to be similar to the previous experiment (data not shown). Description of results is therefore limited to *ex vivo* analysis of the fluorescence. As shown in Figure 5, the *ex vivo* analysis revealed similar fluorescent signal (i.e. intensity over time) in the plasma, urine, kidneys, thymus, small intestine and skin isolated at different time points for mice injected with either stressed or unstressed MSA-Alexa700. However, for the spleen, urinary bladder, liver, heart, lungs, large intestine, brain, muscle and stomach significant differences in distribution of fluorescent signal over time were found. Similar to the first experiment, the highest fluorescent signal was found in the liver at 3 hrs p.i., and this signal was almost 6 times higher in mice treated with the unstressed formulation compared to animals injected with stressed MSA-Alexa700 (1091.4±154.6% ID/g tissue and 209.7±55.8 ID/g tissue, respectively). Accordingly, the distribution over time (p = 0.001) and magnitude of fluorescent signal (p = 0.004) differed in the livers of mice injected with unstressed and stressed MSA-Alexa700.

Although no significant difference was found between the overall fluorescence signals of spleens of mice treated with stressed or unstressed MSA-Alexa700, the distribution of fluorescent signal over time differed between these groups (p = 0.041). Within the first 8 hrs p.i. a higher fluorescent signal was present in the spleens of mice receiving unstressed MSA-Alexa700 compared to mice that received the stressed formulation. At 3 hrs p.i., the fluorescence measured in the spleens of mice injected with unstressed MSA-Alexa700 was 3 times higher than the fluorescence of spleens from animals treated with stressed MSA-Alexa700. However, at 24 hrs p.i. the fluorescence measured in the spleens of mice injected with stressed MSA-Alexa700 was found to be 3 times higher than in spleens of mice injected with unstressed MSA-Alexa700. Moreover, in mice treated with stressed MSA-Alexa700 again “hotspots” with high fluorescence signal were present (like observed in the first experiment).

While in most of organs the fluorescence was increasing until 3 hrs p.i., the signal in the intestines of mice injected with stressed MSA-Alexa700has already reached a maximum 15 min p.i.. Interestingly, the fluorescence in the large intestines of mice treated with both stressed and unstressed MSA-Alexa700 was much higher than in the small intestines.

## Discussion

Presence of protein aggregates in the formulation of therapeutic proteins has been identified as a major risk factor for immunogenicity. However, there is almost no data showing the fate of these aggregates upon injection. It has been hypothesized that aggregates can persist at the site of injection, especially when applied SC or IM, much longer than protein monomers. Therefore aggregates may trigger the activation of immune cells located at the site of injection more efficiently than monomers.

Current experiments were designed to study the biodistribution of unstressed, mainly monomeric, and stressed, mostly aggregated, MSA in mice when applied via different routes. We first showed that metal catalyzed oxidation is an effective way to obtain a MSA formulation containing high amounts of aggregates. Subsequently, we showed that stressed MSA-Alexa700 persists longer at the site of injection compared to unstressed MSA-Alexa700 after SC and IM administration. The time frame, in which the experiment was performed (up to 48 hrs), did not allow for a complete clearance of fluorescence at the injection site, however it could be hypothesized that in a longer experiment these differences between stressed and unstressed MSA Alexa700 treatment would be further magnified. Nonetheless, our observation that stressed MSA-Alexa700 persists longer at the site of injection is in agreement with the hypothesis that aggregates are cleared slower than monomers when given SC. This might explain why for some drugs SC route appears to be the most immunogenic.

High immunogenicity of proteins when injected IV has been described for rhIFNβ [11], PEGylated Factor VIII [12] and antiTNFα antibody [20]. In our previous manuscript we suggested that aggregates, when injected directly into the blood stream, might be rapidly removed by the macrophages of the reticuloendothelial system in the spleen, in a similar manner as in case of liposomes exceeding 100 nm in diameter [18]. In current experiment we induced the formation of aggregates exceeding 100 nm and small, highly fluorescent “hotspots” in the spleens were present. These spots might be aggregates caught by macrophages or might be aggregates that are stuck in the blood vessels. Considering these spots in the spleen and potential importance of macrophage uptake it would be worth performing an in depth study on what causes these “hotspots”, not only for IV administration, but for IP treatment as well.

Another very interesting finding was that “hotspots” were also found in the lungs of mice treated IV with stressed MSA-Alexa700. Since the lungs are composed of very small blood capillaries it appears likely that aggregates might have clogged them. It is possible that macrophages present in the lung tissue take up these particles and therefore increase immunogenicity.

The biodistribution of MSA-Alexa700 upon IP administration differs depending on the amount of aggregates in the formulation. In general, the accumulation of fluorescently labeled and stressed MSA seems lower in the majority of organs compared to accumulation of unstressed MSA. This could be due to a lower diffusion rate of the stressed protein through the peritoneum as well as due to a slower uptake of aggregates by the lymphatic transportation system. A very interesting observation was the longer persistence of fluorescent signal and its inhomogeneous distribution (hotspots) in the spleens of animals injected with stressed MSA-Alexa700. The mechanism, by which these hotspots are formed, is not clear. Their size and number suggests that the origin of “hotpots” in spleens from mice injected IP differs from those found in mice administered IV. IV injection with MSA-Alexa700 induced a high number of small “hotspots”, while IP administration resulted in a low number of “hotspots” with a relatively large surface. Further studies, on what composes these “hotspots” and how they are formed, are therefore needed.

Although the data presented in this manuscript seems to explain some observations described in the literature on how the route of administration affects immunogenicity of protein drugs, one should be aware of the limitations of the employed assays and mouse model. Because we aimed at obtaining an autologous system we have chosen MSA as a protein model. However, as MSA receptors are located in liver and the liver being the main organ of MSA metabolism [21], [22], the elevated fluorescence signal in this organ might not be representative for other proteins. Moreover, choosing a self-protein as a model did not allow direct detection of injected protein by ELISA for example, because of high background signal from endogenous MSA. Therefore the detection of injected MSA could only be done with the use of a label, in this case Alexa-700. A possible issue that could arise is the dye separating from the protein during the degradation of MSA-Alexa700 conjugates in liver, resulting in the detection of free dye and not of the protein itself. We showed that our injected MSA formulations contained less than 5% of free dye, which should not interfere with the detection of the conjugated MSA-Alexa700. However, our data also suggests that MSA-Alexa700 might be degraded quite rapidly *in vivo* (within hours). Because the MSA-Alexa700 conjugates are too big to be filtered out by kidneys, the fluorescence found in the urine most likely originates from MSA-Alexa700 degradation. The fluorescent signal in the urine was high (reaching at maximum almost 3000% of ID/ml), which therefore supports degradation of MSA-Alexa700 conjugates and suggests that the degradation product might differ in spectral properties from conjugates. Release of free dye might potentially lead to an overestimation of MSA-Alexa700 concentration in collected organs and tissues. However, any free dye released by MSA-Alexa700 catabolism in liver would be most probably rapidly removed from the circulation by renal filtration due to the small size of Alexa700. Our data seem to confirm this hypothesis. Moreover, additional SDS-PAGE analysis of selected samples of the livers, spleens, lungs and skin suggested degradation of MSA-Alexa700 in livers but not in other organs (see Appendix S1 and Figure S1). Very high fluorescence was found only in the liver, urinary bladder and urine. Moreover, relatively low signal in plasma suggests rapid removal of free dye from the circulation and therefore low background signal potentially originating from free dye. Thus, any accumulation of fluorescent signal in organs other than liver or urinary bladder is most likely a result of MSA-Alexa700 conjugates present in those organs and not due to the fluorescence of free dye from blood. Moreover, lower fluorescent signal in almost all organs/tissues collected from mice injected IP with stressed MSA-Alexa700 strongly suggests slower and/or less efficient diffusion/lymphatic uptake of the stressed formulation through the peritoneum into the blood circulation. Nevertheless, the most crucial findings described in this manuscript, i.e. i) longer retention of stressed (aggregated) than unstressed MSA-Alexa700 at the site of injection after SC and IM administration, ii) formation of fluorescence “hotspots” in lungs, liver and spleen of mice injected IV or IP with the stressed formulation, iii) lower accumulation of the fluorescent signal in most organs after IP injection of stressed MSA rather than unstressed MSA-Alexa700, can only be explained by differences in biodistribution between stressed and unstressed MSA-Alexa700.

## Conclusions

In this report we show that *in vivo* florescence imaging, despite some drawbacks, is a valuable method to study the biodistribution of protein (aggregates) upon injection. We showed (i) that biodistribution of MSA differs depending on the formulation (stressed or unstressed) and (ii) that the biodistribution of MSA strongly depends on the application route. Furthermore, IV and IP injections of stressed MSA-Alexa700 resulted in the formation of fluorescent “hotspots” in spleens (IP and IV), livers (IP) and lungs (IV), suggesting entrapment of MSA-Alexa700 aggregates in the microenvironment that might enhance induction of antibody responses.

## Supporting Information

Appendix S1 **Testing of potential** ***in vivo*** **degradation of MSA-Alexa700 conjugates. Methodology and results.**
(DOCX)

Figure S1 **SDS-PAGE analysis of samples collected in** ***in vivo*** **experiments 1 (A–C) and 2 (D).** A) Skin samples collected from mice injected SC, B) lungs' samples from mice injected IV, C) Spleen samples from mice injected IP. D) Liver samples collected at different time points in the Study 2.4. US - unstressed MSA-Alexa700, S - stressed MSA-Alexa700. D/ml).
(TIF)
