# Supplementary material for: Influence of Aggregation and Route of Injection on the Biodistribution of Mouse Serum Albumin
Source: PLoS One. 2014 Jan 22;9(1):e85281. doi: 10.1371/journal.pone.0085281 (PMC3898957; doi:10.1371/journal.pone.0085281)
Supplement: Appendix S1 — Testing of potential in vivo degradation of MSA-Alexa700 conjugates. Methodology and results. (DOCX) [file pone.0085281.s001.docx]

**Supplementary data**

1. Material and methods

The following organs’ samples from *in vivo* experiment 1 were analysed i) skin from mice administered SC, ii) lungs from animals injected IV and iii) spleen from animals treated IP. Five organs’ samples from mice treated with unstressed and stressed MSA-Alexa700 were analysed. In addition, liver samples from different time points collected in *in vivo* experiment 2 were tested in order to study potential degradation of MSA-Alexa700 conjugates. Before SDS-PAGE analysis organs/tissues were homogenised in RIPA buffer (the same samples as used for *ex vivo* determination of MSA-Alexa700 biodistribution, see paragraphs *In vivo* experiments 1 and 2). Before analysis the samples were stored at -20˚C. The analysis was performed with the X-Cell Sure Lock^@^ Mini-Cell system (Invitrogen, the Netherlands). A volume of 20 µl of homogenized samples were loaded on the NuPAGE^®^ 4-12% Bis-Tris Mini Gels of 1,5 mm thickness (Novex^®^, Invitrogen, the Netherlands). Samples from experiment 1 were separated under non-reducing conditions at 130 V for 60 minutes. Due to high viscosity, homogenized livers from experiment 2 were separated under reducing conditions at 130V for 60 minutes. Presence of conjugates was visualized by detection of fluorescence using the infrared imager Odyssey (LiCore, Germany).

**Results and discussion**

Figure S1 shows the results of the SDS-PAGE analysis of samples collected in experiment 1 (Figure S1. A-C) and experiment 2 (Figure S1. D). In 4 out of 5 mice injected SC with stressed MSA-Alexa700 the fluorescent signal of skin samples was much higher than in samples from mice injected with unstressed MSA-Alexa700 (Figure S1. A). Moreover, in skin samples from mice injected with stressed formulation, bands of very high molecular weight proteins/aggregates, too big to enter the gel, were found. This suggests the presence of intact MSA-Alexa700 aggregates at the site of injection at 48hrs p.i. Bands indicating MSA-Alexa700 aggregates too big to enter the gel were also found in lungs’ samples of mice injected IV with stressed MSA-Alexa700 (Figure S1. B). These bands might represent big aggregates observed as fluorescent “hotspots” in the biodistribution experiment. In contrast, in samples from mice injected with unstressed MSA-Alexa700 this fraction of high molecular weight was almost undetectable. Similarly, SDS-PAGE analysis of spleen homogenates from animals injected IP with stressed MSA-Alexa700 revealed several fluorescent bands corresponding to conjugates with higher molecular weight than monomeric MSA-Alexa700 (Figure S1. C). In all samples from mice injected with unstressed MSA-Alexa700 a clear band corresponding to monomeric MSA-Alexa700 was detected. Bands corresponding to higher molecular weight species of MSA-Alexa700 were not detectible.

To test the hypothesis that *in vivo* MSA-Alexa700 degradation could have happened *in vivo,* liver samples from experiment 2 were analysed. Figure S1. D shows the results of SDS-PAGE analysis. The monomeric MSA-Alexa700 could be found in the livers of mice IP injected with unstressed MSA-Alexa700 already 15min p.i. and was detectible up to 3 hrs p.i.. However, most of the MSA-Alexa700 conjugates were likely degraded between 1 and 3 hrs p.i. as the amount of MSA-Alexa monomers at the latter time point was greatly decreased. No MSA-Alexa700 conjugates were observed 8 and 24 hrs p.i. in samples from mice receiving unstressed MSA-Alexa700. In contrast, in mice IP injected with stressed MSA-Alexa700 the fluorescent signal of bands of high molecular weight species could be detected until the end of the experiment (24hrs p.i.). Moreover, despite the fact that clear bands corresponding to MSA-Alexa700 monomers could be measured in samples collected 15 min and 1hr p.i., the overall fluorescent signal seemed to be similar for all samples collected at different time points during the biodistribution study. However, the overall fluorescent signal seemed to be reduced when compared to the liver samples from mice injected with unstressed MSA-Alexa700 formulation.

The data obtained with SDS-PAGE analysis seems to confirm the conclusions presented in the manuscript. In organs collected from mice injected with stressed MSA-Alexa700 formulation 48hrs p.i. (experiment 1) a strong signal was found for MSA-Alexa700 conjugates compared to samples from animals injected with unstressed formulation. Analysis of liver samples from study 2 confirmed fast degradation of conjugates in livers. Interestingly, unstressed MSA-Alexa700 seemed to be more prone to degradation than stressed MSA-Alexa700. SDS-PAGE also confirmed that the fluorescence signal of the conjugate degradation products most likely has different spectral properties (higher fluorescence) compared to intact conjugates. The fluorescence of dye released in livers (time points 3-24hrs p.i.) was much higher than fluorescence of conjugates accumulated in livers (time points 15min p.i. and 1hrs p.i.). That might have influenced the outcome of the biodistribution study as MSA-Alexa700 accumulation might have been overestimated. However, as intact conjugates were found in all samples collected 48 hrs p.i., possible overestimation of MSA-Alexa700 accumulation seems to be an issue only for liver, urinary bladder and urine samples (please see paragraph 4).

One has to keep in mind that described SDS-PAGE analysis has been performed on samples stored for ~ 12 months in RIPA buffer at -20˚C. However, the stability of MSA-Alexa700 conjugates under these conditions is unknown. Free dye found in SDS-PAGE analysis could have originated (partly) in the process of MSA-Alexa700 degradation during storage. The impact of SDS-PAGE conditions on old, stored samples is also unknown. It also cannot be excluded that conjugates were separated and free dye originated (partly) due to the SDS-PAGE procedure itself. Nonetheless, data obtained in SDS-PAGE analysis seems to confirm that i) stressed MSA-Alexa700 is retained longer at the site of injection than unstressed MSA-Alexa700, ii) stressed MSA-Alexa700 seems to accumulate in lungs of mice injected IV and spleens of mice injected IP, iii) liver seems to be the main organ, in which MSA-Alexa700 conjugates are degraded.
