# Supplementary figures and images for: Influence of Aggregation and Route of Injection on the Biodistribution of Mouse Serum Albumin
Source: PLoS One. 2014 Jan 22;9(1):e85281. doi: 10.1371/journal.pone.0085281 (PMC3898957; doi:10.1371/journal.pone.0085281)

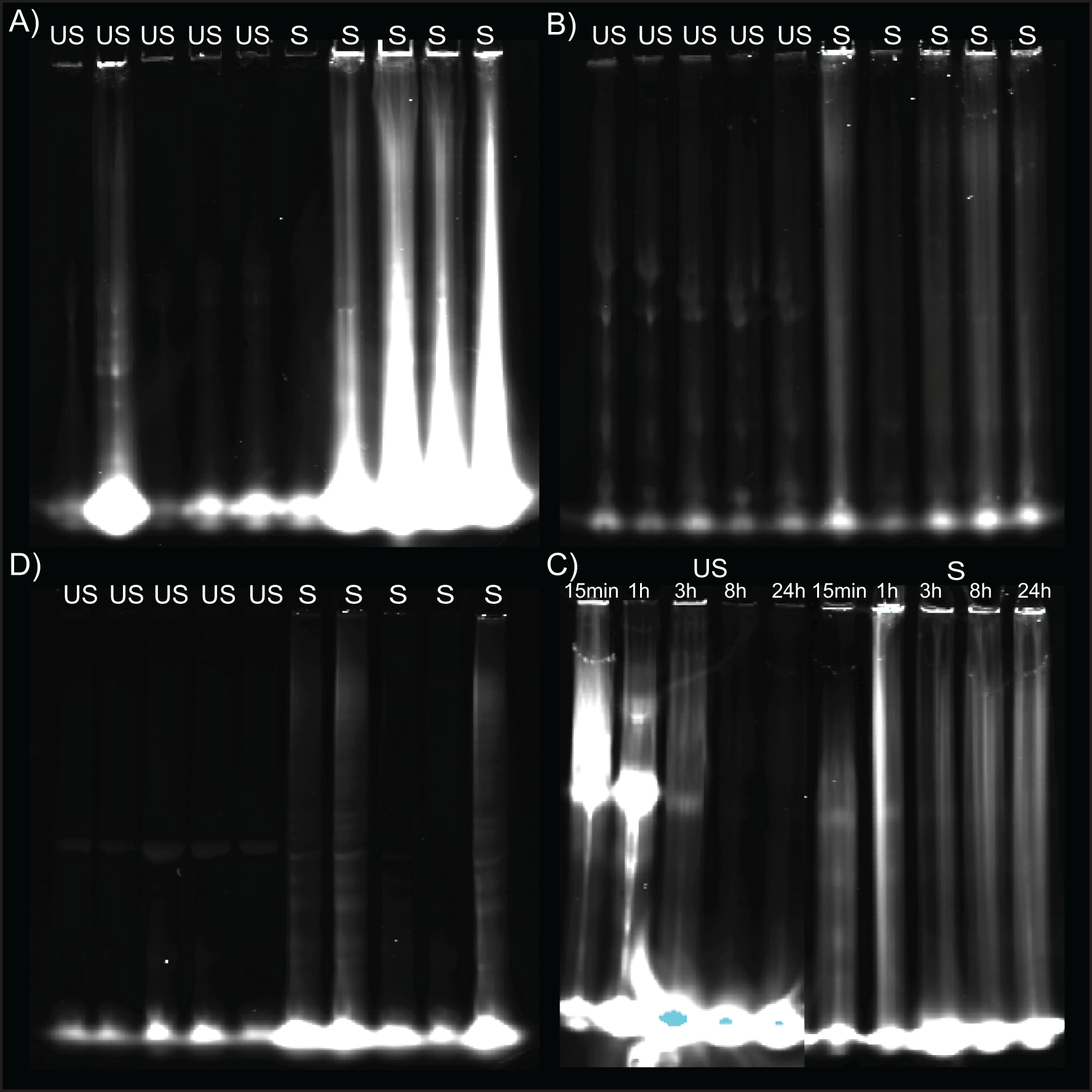

Supplement: Figure S1 — SDS-PAGE analysis of samples collected in in vivo experiments 1 (A–C) and 2 (D). A) Skin samples collected from mice injected SC, B) lungs' samples from mice injected IV, C) Spleen samples from mice injected IP. D) Liver samples collected at different time points in the Study 2.4. US - unstressed MSA-Alexa700, S - stressed MSA-Alexa700. D/ml). (TIF) [file pone.0085281.s002.tif]
